# Management of peri-implantitis using local drug delivery among Indian patients

**DOI:** 10.6026/973206300191301

**Published:** 2023-12-31

**Authors:** Shilpi Khare, Hitika P Doda, Vanya Dubey, Shreyansh Sutaria, Shib Kumar Nath, Drishti Bhatt, Santosh Kumar, Prachi Desai

**Affiliations:** 1 Department of Prosthodontics, Bhabha College of Dental Sciences, Bhopal, M.P., India; 2 Midtown Dental Group, New Jersey, USA; 3 Department of Oral and Maxillofacial Surgery, Rungta College of Dental Sciences and Research, Kohka Kurud, Bhilai, Chhattisgarh, India; 4 Department of Oral Medicine and Radiology, Gujarat University, Gujarat, India; 5 Department of Orthodontics, The Smile Architect Dental Clinic and Braces Centre, Agartala, Tripura, India; 6 Department of Oral and Maxillofacial Surgery, Divya Jyoti College of Dental Sciences and Research, Modinagar, Uttar Pradesh, India; 7 Department of Periodontology, Karnavati School of Dentistry, Karnavati University, Gandhinagar, Gujarat, India; 8 Oral Pathology and Microbiology, Ahmedabad Dental College and Hospital, Ahmedabad, India

**Keywords:** Local drug delivery, chlorhexidine, peri-implantitis

## Abstract

It is of interest to compare 0.2% chlorhexidine gel, 0.2% chlorhexidine chip, minocycline microspheres and slow-release doxycycline gel and tetracycline fibers as drug delivery systems in the management of peri-implantitis. The study comprised of 105 Indian participants who had a minimum of one dental implant with a probing depth of 4 mm, along with exudate and/or bleeding upon probing along with the presence of potentially harmful germs. The use of minocycline microspheres and 0.2% chlorhexidine gel resulted in significant improvements in probing depths at 1 month, 3 months and 6 months and all treatments showed decline in the indicator bacteria. Thus, minocycline microspheres and 0.2% chlorhexidine gel is useful as an adjuvant for mechanical debridement in management of peri-implantitis.

## Background:

Osseointegrated dental implants are utilised extensively since the first patient was operated upon by Brånemark in 1965 [1,2]. Dental implants possess 20-year survival rates that are about 96% [3,4]. The overall success effectiveness of dental surgical implants is 89.7 percent at an average follow-up duration of 15.7 years. Despite their high longevity and achievement rates, osseo-integrated surgical implants can experience biological difficulties, i.e. peri-implant ailments [5, 6]. There is an incidence of peri-implantitis, or inflammatory processes of the peri-implant mucosa with advancing bone loss, to be 12.8% at the level of dental implant and 18.5% at the patient's level [7, 8]. However, the frequency at the patient level varies between 1% to 47% [9, 10].

Globally, more than 12 million implants are installed annually and peri-implantitis develops each year in over 1 million dental implants [11,12]. The management of peri-implantitis follows the standard procedure of periodontitis management due to the complexity of the microbiota involved with the condition, which includes numerous pathogenic microorganisms [13,14]. Treatment options for peri-implantitis comprise anti-microbial therapy, photo-dynamic therapy, laser-assisted debridement, non-surgical debridement, open flap debridement, air abrasion, guided bone regeneration with or without bone transplants and supportive therapy [15,16]. Numerous locally administered anti-microbials, including tetracycline fibres, 0.2% chlorhexidine gel, chlorhexidine chips, minocycline microspheres, and gradual-release of doxycycline gel have been utilised as an adjuvant to non-surgical cleansing in the treatment of peri-implantitis [17, 18]. Systematic reviews [19,20] suggest that there is not enough evidence to recommend the use of supplemental antimicrobial medication for the management of peri-implantitis. Therefore, it is of interest to compare 0.2% chlorhexidine gel, 0.2% chlorhexidine chip, minocycline microspheres, and slow-release doxycycline gel and tetracycline fibers as drug delivery systems in management of peri-implantitis.

## Methods and Materials:

The study comprised of 105 Indian participants who had a minimum of one dental implant with a probing depth of 4 mm, along with exudate and/or bleeding upon probing and with the presence of potentially harmful germs. Participants were allocated at random to receive 0.2% chlorhexidine gel (21 patients, 45 implants), 0.2% chlorhexidine chip (20 patients, 41 implants), minocycline microspheres (22 patients, 48 implants), slow-release doxycycline gel (21 patients, 45 implants) and tetracycline fibers (21 patients, 43 implants) following debridement. Treatments were carried out at three timeframes: baseline, one month and 3 months and follow-up evaluations were conducted at 10 days and at 1 month, 3 month, 6 month, 9 month, and 12 months.

The study excluded individuals who had any of the following conditions:

[1] Females who were pregnant, nursing, or of childbearing potential who were not using appropriate birth control techniques;

[2] Medicine containing substances known to alter periodontal health during a month following the screening visit;

[3] The need for preventative antibiotics in treatment;

[4] Systemic antibiotic use in the three months prior to the study, and

[5] Sensitivity to tetracyclines.

## Measurements:

All of the measurements were taken by one examiner, who was not informed of the patient's intervention group at the time of screening, 10 days and at 1 month, 3 month, 6 month, 9 month, and 12 months.

## Complete-mouth plaque score:

Dental plaque across the gingival/mucosal border measured following the application of a disclosing dye and reported as a percentage of each patient's studied sites (a total of six locations per tooth as well as dental surgical implant).

## Complete mouth bleeding score:

Bleeding that becomes apparent after the probing depth is measured and is reported as a percentage of the sites that were tested (a total of six locations every tooth and dental surgical implant).

## Local plaque score:

The percentage of implant areas in each patient that have dental plaque across the mucosal border at four locations on every treated implant was determined following the application of a revealing dye.

## Probing depth:

Each medicated implant's four locations were measured, to the nearest measurement, employing a plastic probe and a 0.2 N standard force.

## Microbial sampling:

Cotton pieces were used to isolate each qualified implant's deepest spot. Sterilised cotton pellets were used to remove supragingival plaque. For twenty seconds, four submucosal paper points were placed and held there until resistance was overcome. Using sterile cissors, the pointed ends of two paper points were removed. The vial holding 3.3 ml of decreased transport fluid VMGA III was then utilised for microbiological culture. To be employed with the DNA method, the remaining two paper tips were put in an uncontaminated, dry Eppendorf tube.

## Treatment:

Along with instructions on oral hygiene, supra- and subgingival calculus and plaque were removed from implant surfaces using a rubber cup with polishing paste and scalers made specifically for implants (Hawe Neos deplaquer, Hawe Neos dental, Switzerland). Patients were randomized to receive treatment with any drug delivery system of 0.2% chlorhexidine gel, 0.2% chlorhexidine chip, minocycline microspheres, and slow-release doxycycline gel and tetracycline fibers. Numbered sealed envelopes containing cards designating the supplementary usage of 0.2% chlorhexidine gel, 0.2% chlorhexidine chip, minocycline microspheres, slow-release doxycycline gel and tetracycline fibers were inserted at random. The treating physician opened the envelopes and gave the patient the prescribed medication. The medication was not disclosed to the examining doctor, who was in charge of documenting clinical parameters and collecting microbiological samples.

## Data analysis and statistics:

Applying independent sample t- statistical tests for continuous data variables and Fisher's exact statistical analyses for categorical factors, the five treatment groups were compared at screening with regard to different features. In terms of plaque score (percentage), probing depths assessments, and bleeding on probing (percentage), the consequences of treatment were evaluated. For the microbiology data acquired using checkerboard examination, a mean value was computed for the various bacteria of every patient. The number of CFU from anaerobic, non-selective Brucella blood agar (BRU) plates was used to estimate TVC. Enteric rods, enterococci, and black-pigmented anaerobic microbes (P. nigrescens, P. intermedia and P. gingivalis) were estimated as a percentage of TVC.

## Results:

The mean age of study participants treated with 0.2% chlorhexidine gel, 0.2% chlorhexidine chip, Minocycline microspheres, Doxycycline gel and Tetracycline fibers was 62.2 ± 9.7 years, 66.7± 9.7 years, 62.2 ± 9.7 years, 66.7 ± 8.9 years and 62.2 ± 9.7 years, respectively. There were 12 female and 9 male in 0.2% chlorhexidine gel group, 10 males and 10 females in 0.2% chlorhexidine chip group, 10 females and 12 males in Minocycline microspheres group, 11 female and 10 male in Doxycycline gel group and 12 female and 09 male in tetracycline fibers group. Smoking history revealed following information: 10 study participants never smoked, 03 study participants were former smoker while 08 were present smokers in 0.2% chlorhexidine gel group. 09 never smoked 04 were former smoker while 07 were present smokers in 0.2% chlorhexidine chip group. 10 never smoked, 03 were former smoker while 08 were present smokers in Minocycline microspheres group. 09 never smoked, 03 were former smoker while 09 were present smokers in Doxycycline gel group. 08 never smoked, 04 were former smoker while 09 were present smokers in Tetracycline fibers group. There was no specific difference between the demographic properties of study participants in different categories (Table 1). Table 2 shows the average plaque scores at baseline, 10 days, 1 month, 2 months, 3 months and 6 months, 9 months and 12 months. There is a gradual decrease in plaque scores from baseline to 3 months and it increased after 3 months. Highest plaque scores were seen at 6 months in 0.2% chlorhexidine chip group with 32±04. Table 3 shows significant reduction in mean probing depth from screening up to 12 month follows up (4.1±0.4 mm to 3.7±0.6 mm). There was significant reduction in mean probing depth from screening up to 12 month follow up (4.1±0.7 mm to 3.9± 0.1mm). It was found that use of minocycline microspheres and 0.2% chlorhexidine gel as compared to other drug delivery system resulted in significant improvements in probing depths at 10 days 1 month, 3 month, and 6 months. There was significant reduction in probing depth in each local drug delivery system in overall 12 month follow up. Table 4 shows significant reduction in bleeding score in overall 12 month follow up (91±12%, to 81±03%). It was found that use of minocycline microspheres and 0.2% chlorhexidine gel resulted in significant improvements in bleeding scores at 10 days 1 month, 3 month, and 6 months. There was significant reduction in bleeding scores in each local drug delivery system in overall 12 month follow up. The mean values for every bacterial species under investigation peaked at visit one and progressively dropped over the course of the trial. For any pathogen and at any period, there was no statistically significant difference seen between all antimicrobials. After six to twelve months in culture, the TVC increased significantly and peaked at 106 cells/ml of transport medium. Each of the five groups saw a similar increase. After treatment, P. gingivalis was found to have significantly decreased in all groups. During the course of the one-year study period, P. gingivalis was found to have remained at very low levels in the minocycline group and 0.2% chlorhexidine gel (<0.2% of TVC). On other cases, enteric rods as well as enterococci were found, but in small amounts of TVC. Enteric rods, however, exhibited a slightly and momentarily greater amount (not statistically meaningful) in five the groups receiving treatment at third visit (after the antibiotic therapy).

## Discussion:

Better outcomes in bleeding on probing and periodontal probing depths in peri-implantitis using locally delivered antimicrobials. However, current reviews show that there is not enough evidence to recommend the use of supplemental antimicrobial medication for the management of peri-implantitis. It was found that the use of minocycline microspheres and 0.2% chlorhexidine gel as compared to other drug delivery system resulted in significant improvements in probing depths at 10 days 1 month, 3 month, and 6 months. According to prior studies, minocycline increases the likelihood of bacterial resistance by reducing inflammatory cytokines, which results in considerable reductions in PD and BOP. However, regular treatments are required to sustain the therapeutic outcomes [16-20]. Minocycline had effective antibacterial efficacy when combined with metronidazole [21-22,23]. After combining the data from several included studies regarding the effectiveness of minocycline, we found that metronidazole, which had demonstrated a favorable antimicrobial effect against a variety of microorganisms, was more likely to be the cause of these results than the immediate consequence of minocycline [18-21]. Minocycline introduced as an ointment in the surgical process did not show substantial advantages when compared with a placebo ointment [16-19]. It has been documented that tetracycline used topically significantly improves clinical measures like PD, clinical attachment level (CAL), and the sulcular bleeding index (SBI) [16-24]. Moreover, applying topical or local antibiotics at the area of surgery may help to reduce post-implant inflammation [12,13, 17,18,21]. A study [16-17] documented better outcomes in the microbiological and clinical variables when tetracycline was used topically to treat peri-implantitis. Few comparison studies show that reductions in the degree of inflammation in the mucous membrane and probing depth in early peri-implant lesions after mechanical or combination of mechanical and antimicrobial intervention have been achieved in the past [21- 25]. A number of contrasting conclusions also surfaced. Few previous studies were unable to show a discernible difference between the groups receiving treatment and those receiving no treatment [26-27]. On the other hand, few studies found that integrating non-surgical peri-implantitis management with locally administered minocycline and chlorhexidine irrigation led to substantial improvements in clinical as well as radiographic parameters [27- 28]. Studies are showing that clinical as well as radiographic parameters were enhanced by a straightforward non-surgical technique that combined intra-sulcular chlorhexidine with local administration of minocycline [28]. The divergent results could be attributed to local/topical antibiotics' inability to reach a significant amount on the implant surface [16- 18]. This challenge might deter medical professionals from using topical or local antibiotics. Clinicians must use more local/topical antibiotics overall in order to attain a high concentration of these drugs, which raises the possibility of activity of antimicrobial resistance and unfavorable side effects [19- 20]. The mean values for every bacterial species under investigation peaked at visit one and progressively dropped over the course of the trial. For any pathogen and at any period, there was no statistically significant difference seen between all antimicrobials. After six to twelve months in culture, the TVC increased significantly and peaked at 106 cells/ml of transport medium. Each of the five groups saw a similar increase. After treatment, P. gingivalis was found to have significantly decreased in all groups. During the course of the one-year study period, P. gingivalis was found to have remained at very low levels in the minocycline microspheres group and 0.2% chlorhexidine gel (<0.2% of TVC). On other cases, enteric rods as well as enterococci were found, but in small amounts of TVC. Enteric rods, however, exhibited a slightly and momentarily greater amount (not statistically meaningful) in five the groups receiving treatment at third visit (after the antibiotic therapy). If a more sensitive technique or more patients with severe infections had been chosen, it's probable that we might have seen more noticeable microbiological consequences of the treatment. The low TVC measured at the starting point of the investigation validates the low bacterial burden at that time. If samples are taken from failing implants, it's also feasible that a different sampling technique needs to be applied. When compared to a selective culture procedure, the tip of the paper point may only collect a small fraction of the peri-implantitis lesion's bacterial flora, increasing the chance of false-negative samples. A study shows that when minocycline microspheres were used locally as part of the CIST regimen for the treatment of peri-implantitis, substantial improvements in clinical as well as microbiologic parameters were obtained after three months [29]. Our results suggest that topical antibiotic treatments may lessen pocket depth (PD), although their effectiveness seems to be time-limited. Studies has demonstrated the efficacy of using minocycline-containing microspheres as an adjuvant to mechanical treatment for periodontal as well as peri-implantitis lesions [22,25]. The current study's enhanced results when compared to the usage of supplementary chlorhexidine gel indicated the usefulness for peri-implant lesions as well. Data also suggest that the clinical benefits of using minocycline microspheres can last for a full year. However, it is still unclear to what degree the combined mechanical and minocycline therapy could be deemed sufficient for the lesion that has been treated.

## Conclusion:

Data shows that minocycline microspheres and 0.2% chlorhexidine gel as local drug delivery system is useful as adjuvants to mechanical debridement in management of peri-implantitis.

## Figures and Tables

**Table 1:** Demographic details

**Participant's Characteristics**	**Mean Age ± SD (years)**	**Gender (female/male)**	**Smoker (never/former/present)**	**Number of treated implants**
0.2% chlorhexidine gel	62.2±9.7	12-Sep	10-03-2008	45
0.2% chlorhexidine chip	66.7± 9.7	10-Oct	09-04-2007	41
Minocycline microspheres	62.2±9.7	10-Dec	10-03-2008	48
Doxycycline gel	66.7±8.9	11-Oct	09-03-2009	45
Tetracycline fibers	62.2 ±9.7	12-Sep	08-04-2009	43

**Table 2:** Average plaque scores ± standard deviation (%) for each of the four implant locations at treated implants throughout the course of a 12-month observation period

	**Screening**	**10 days**	**1 month**	**2 months**	**3 months**	**6 months**	**9 months**	**12 months**
0.2% chlorhexidine gel	50±18	45±15	16±08	23±10	23±02	30±11	22±09	27±18
0.2% chlorhexidine chip	51±27	46± 19	21±09	27±13	27±04	32±04	27±10	21±04
Minocycline microspheres	50±19	43±17	14±03	23±06	23±02	30±07	23±11	21±06
Doxycycline gel	51±15	46± 16	20±06	27±11	27±03	31±14	27±03	27±14
Tetracycline fibers	50±21	45±14	21±04	27±09	23±11	31±17	26± 10	27±13

**Table 3:** Average probing depths ± standard deviation (mm) for treated implants at each of the four locations over the course of the 12-month observation period

	**Screening**	**10 days**	**1 month**	**2 months**	**3 months**	**6 months**	**9 months**	**12 months**
0.2% chlorhexidine gel	4.1±0.4	3.9±0.3	3.8±0.7*	3.7±0.7	3.7±0.6*	3.8±0.6*	3.8±07	3.7±0.6
0.2% chlorhexidine chip	4.1±0.7	4.0±0.7	3.9±0.3	4.0±0.3	3.9±0.3	3.9±0.4	3.9±0.2	3.9±0.1
Minocycline microspheres	4.1±0.3	3.9±0.3	3.8±0.8*	3.6±0.8	3.8±0.8*	3.7±0.7*	3.9±0.8	3.8±0.5
Doxycycline gel	4.1±0.7	4.0±0.7	3.9±0.2	4.0±0.2	4.0±0.2	3.9±0.3	3.9±0.2	3.9±0.1
Tetracycline fibers	4.1 ±0.7	4.0±0.7	3.8±0.1	3.9±0.2	3.9±0.2	3.8±0.3	3.8±0.4	3.8±0.2
*represent statistically significant difference (p<0.05)

**Table 4:** Average bleeding on probing/microbial sampling scores ± SD for treated implants at each of the four implant sites throughout the course of a 12-month observation period

	**Screening**	**10 days**	**1 month**	**2 months**	**3 months**	**6 months**	**9 months**	**12 months**
0.2% chlorhexidine gel	89±06	62±24	46±16*	44±21*	51±26*	61±25*	68±02	77±22
0.2% chlorhexidine chip	91±12	72±14	66±12	65±08	74±11	82±09	75±12	81±03
Minocycline microspheres	89±07	63±21	47±13*	45±19*	52±18*	62±15*	69±21	78±12
Doxycycline gel	90±05	73±11	67±09	66±18	74±05	82±13	75±09	81±10
Tetracycline fibers	91±13	74±08	68±07	67±12	75±11	82±04	75±11	81±07
